# Retraction: Explainable AI for time series prediction in economic mental health analysis

**DOI:** 10.3389/fmed.2026.1862384

**Published:** 2026-04-27

**Authors:** Frontiers Editorial Office

The publisher retracts the 26 June 2025 article cited above.

Following publication, concerns were raised regarding the validity of the data in the article. The authors failed to provide a satisfactory explanation during the investigation, which was conducted in accordance with Frontiers' policies. Given the concerns, the editors no longer have confidence in the findings presented in the article.

This retraction was approved by the Chief Editor of Frontiers in Medicine and the Chief Executive Editor of Frontiers. The authors received a communication regarding the retraction and had a chance to respond. This communication has been recorded by the publisher.

